# Bismuth-Antimony Alloy Nanoparticles Embedded in 3D Hierarchical Porous Carbon Skeleton Film for Superior Sodium Storage

**DOI:** 10.3390/molecules28186464

**Published:** 2023-09-06

**Authors:** Jiafan Wang, Yonghui Lin, Wei Lv, Yongfeng Yuan, Shaoyi Guo, Weiwei Yan

**Affiliations:** 1 College of Machinery Engineering, Zhejiang Sci-Tech University, Hangzhou 310018, China; 2 Zhejiang Ecowell Energy Management Technology Co., Ltd., Hangzhou 310012, China; 3 Changshan Research Institute, Zhejiang Sci-Tech University, Changshan 324299, China; 4 College of Metrology and Measurement Engineering, China Jiliang University, Hangzhou 310018, China

**Keywords:** sodium-ion batteries, Bi-Sb alloy, carbon, film

## Abstract

A composite film that features bismuth–antimony alloy nanoparticles uniformly embedded in a 3D hierarchical porous carbon skeleton is synthesized by the polyacrylonitrile-spreading method. The dissolved polystyrene is used as a soft template. The average diameter of the bismuth–antimony alloy nanoparticles is ~34.5 nm. The content of the Bi-Sb alloy has an impact on the electrochemical performance of the composite film. When the content of the bismuth–antimony alloy is 45.27%, the reversible capacity and cycling stability of the composite film are the best. Importantly, the composite film outperforms the bismuth–antimony alloy nanoparticles embedded in dense carbon film and the cube carbon nanobox in terms of specific capacity, cycling stability, and rate capability. The composite film can provide a discharge capacity of 322 mAh g^−1^ after 500 cycles at 0.5 A g^−1^, 292 mAh g^−1^ after 500 cycles at 1 A g^−1^, and 185 mAh g^−1^ after 2000 cycles at 10 A g^−1^. The carbon film prepared by the spreading method presents a unique integrated composite structure that significantly improves the structural stability and electronic conductivity of Bi-Sb alloy nanoparticles. The 3D hierarchical porous carbon skeleton structure further enhances electrolyte accessibility, promotes Na^+^ transport, increases reaction kinetics, and buffers internal stress.

## 1. Introduction

Efficient and low-cost energy storage technology is one of the most important and fundamental technologies in modern society. Although lithium-ion batteries (LIBs) have been widely used, the high cost and scarcity of lithium have limited the further development of LIBs in the future. Sodium-ion batteries (SIBs) are considered a competitive alternative to LIBs because of the natural abundance and low cost of sodium (Na) [1,2,3,4]. Many efforts have been devoted to developing the electrode materials of SIBs, particularly anode materials [5,6,7,8,9]. Various carbonaceous materials and transition metal oxides have been studied as anode materials for SIBs [10,11,12]. However, their limited interstitial sites result in low capacity, about 200~300 mAh g^−1^. The growing demand for high-performance SIBs is driving the development of advanced anodes with long life, high reversible capacity, and excellent rate performance [13,14].

Metals such as bismuth (Bi) and antimony (Sb) can store Na ions in a highly reversible alloying manner. This results in a low sodiation potential (<1.0 V vs. Na/Na^+^) and high theoretical capacity (385 and 660 mAh g^−1^) [15,16,17]. Therefore, the two metals are recognized as potential anode materials for SIBs. Unfortunately, during the sodiation/desodiation process, the volume of Bi and Sb significantly varies. This leads to severe pulverization and capacity decline, posing major challenges to Bi and Sb [18,19]. In recent years, bimetallic alloys have received much attention as a feasible strategy. The Bi-Sb alloy combines the relatively small volume expansion of Bi with the high theoretical capacity of Sb [20,21]. The synergistic effect can make up for each material’s shortcomings and achieve a balance of electrochemical performance. Bi and Sb belong to the same main group in the periodic table of elements. The identical atom arrangement induces similar physical and chemical properties. Bi and Sb can be alloyed in any molar ratio, so Bi-Sb alloy allows for flexible composition design. However, like other alloy-type anode materials, Bi-Sb alloy still suffers from a rapid capacity decline due to a volume change of 250–390% during repeated alloying/dealloying processes [22,23,24,25], especially at high current densities. Additionally, the electronic conductivity of Bi is relatively poor, further limiting the rate performance of Bi-Sb alloy. These challenges hinder the practical application of Bi-Sb alloy in SIBs.

To increase the cycle durability and rate performance of Bi-Sb alloy, engineering methods such as nanostructure design and coupling carbon materials have been proposed [26,27,28,29,30]. For example, X.L. Hu et al. prepared Bi-Sb alloy@carbon nanofibers using the electrospinning method. The discharge capacity reached 233 mAh g^−1^ at 2 A g^−1^ [31]. Y.L. Ding et al. synthesized Bi-Sb alloy/N-doped porous carbon. The composite exhibited a discharge capacity of 245 mAh g^−1^ at 2 A g^−1^ [32]. J. Lu et al. electrodeposited triangular pyramid arrays of Bi-Sb alloy on Cu substrate and obtained a reversible capacity of 335 mAh g^−1^ at 2.5 A g^−1^ [33]. S.P. Wu et al. synthesized Bi-Sb alloy nanoparticles confined in tremella-like carbon microspheres for potassium-ion batteries [34]. Most of these works focus on hybridizing Bi-Sb alloy with carbon. However, this strategy still faces challenges. To buffer the volume expansion of Bi-Sb alloy, the composite structure needs to have high strength and limited Bi-Sb content. To promote the diffusion/transfer of Na ions, the composite structure requires high electrolyte accessibility and short Na^+^ diffusion paths. To prevent molten Bi from flowing out due to its low melting point, Bi and Sb need to be effectively sealed inside the composite. It is difficult to achieve all of these objectives simultaneously. This requires an in-depth understanding of the composite structure and the properties of Bi-Sb alloy. Unfortunately, the research on the composite structure of Bi-Sb alloy is very limited.

Herein, Bi-Sb alloy nanoparticles are uniformly embedded into 3D hierarchical porous carbon skeleton films using dissolved polystyrene (PS) as a soft template and polyacrylonitrile (PAN) as a film-forming material (denoted as BiSb@CSF). The effect of Bi-Sb alloy content on the Na storage performance of composite film is investigated. Furthermore, Bi-Sb alloy nanoparticles are also embedded into a dense carbon film using a modified method based on dissolved PS (denoted as BiSb@DCF) and confined in the wall of a cube carbon nanobox by the NaCl template-assisted method (denoted as BiSb@CCB). The effect of the composite structures on the electrochemical performance of Bi-Sb alloy is investigated. It is found that when the content of Bi-Sb alloy reaches 45.27%, BiSb@CSF exhibits a high and stable Na storage performance, affording a remarkable reversible capacity of 185 mAh g^−1^ at 10 A g^−1^ after 2000 cycles and a low capacity decay rate of 0.02% per cycle. This performance surpasses that of BiSb@DCF and BiSb@CCB. The exceptional electrochemical performance of BiSb@CSF is attributed to its unique integrated structure. The robust 3D carbon skeleton and hierarchical pores effectively accommodate the volume variation of Bi-Sb alloy nanoparticles, facilitate the transport of ions and electrons, and accelerate the reaction kinetics. Consequently, BiSb@CSF emerges as an excellent anode material for SIBs.

## 2. Results and Discussion

**Figure 1:**
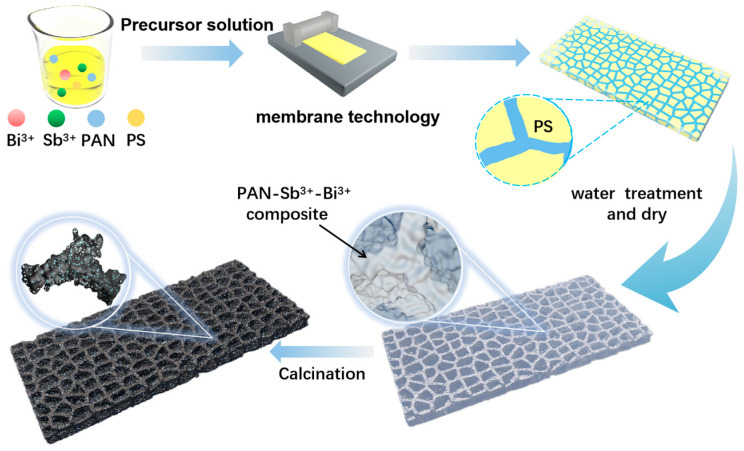
Schematic of synthesis process of BiSb@CSF.

The synthesis procedure for BiSb@CSF is illustrated in Figure 1. DMF, an aprotic polar solvent, is known for its solubility to both organic compounds (PS, PAN) and inorganic compounds (Bi(NO_3_)_3_, SbCl_3_). However, the dissolved states of the four compounds in DMF are different. PS exists in the form of colloidal particles when dissolved in DMF, which allows it to act as soft template due to its independence in DMF. Nevertheless, Bi(NO_3_)_3_, SbCl_3_, and PAN can be completely dissolved in DMF to form a uniform mixed solution. Due to the appropriate viscidity of PAN solution, the PAN-PS-Bi(NO_3_)_3_-SbCl_3_ solution can be spread onto a glass plate to form a continuous film. Upon contact with water, the PAN solution solidifies immediately, rapidly encapsulating the Bi(NO_3_)_3_ and SbCl_3_ within the PAN. At the same time, the PS soft template in the film is washed away, resulting in the formation of hierarchical pores. Consequently, a 3D hierarchical porous PAN skeleton film embedded with Bi(NO_3_)_3_ and SbCl_3_ is constructed. After calcination, this structure is transformed into BiSb@CSF. On the other hand, when the PAN-PS-Bi(NO_3_)_3_-SbCl_3_ precursor film is directly dried, the Bi(NO_3_)_3_ and SbCl_3_ are also encapsulated inside the dried PAN, but there are no 3D hierarchical pores in the film. This is due to the low compatibility of PS in DMF, so the slippery PS colloidal particles easily separate from the PAN-Bi(NO_3_)_3_-SbCl_3_ solution. As a result, Bi-Sb alloy nanoparticles are embedded in a dense film.

The synthesis process of the precursor film is shown in Figure S1. Figure S1a illustrates the PAN-PS-Bi(NO_3_)_3_-SbCl_3_ solution coated on a glass plate. The dissolved PS is evenly mixed in the solution film. Subsequently, when the solution film is washed with water, it rapidly solidifies into a honeycomb porous film, and the dissolved PS is removed (Figure S1b). After drying, the PAN-Bi(NO_3_)_3_-SbCl_3_ precursor film displays a distinct porous skeleton structure (Figure S1c). In contrast, when the PAN-PS-Bi(NO_3_)_3_-SbCl_3_ solution is directly dried to form a film, the precursor film is dense. Pores and skeletons do not appear (Figure S1d).

**Figure 2:**
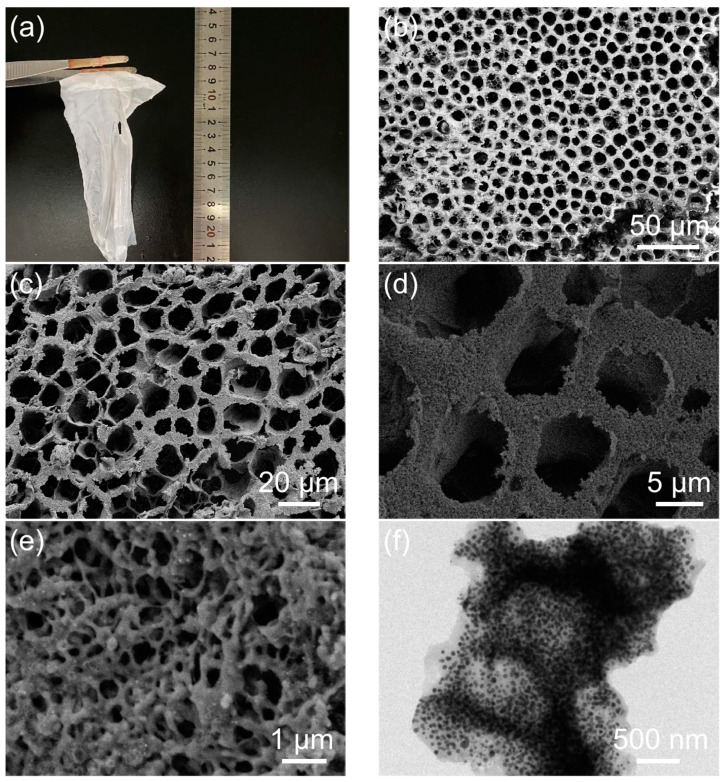

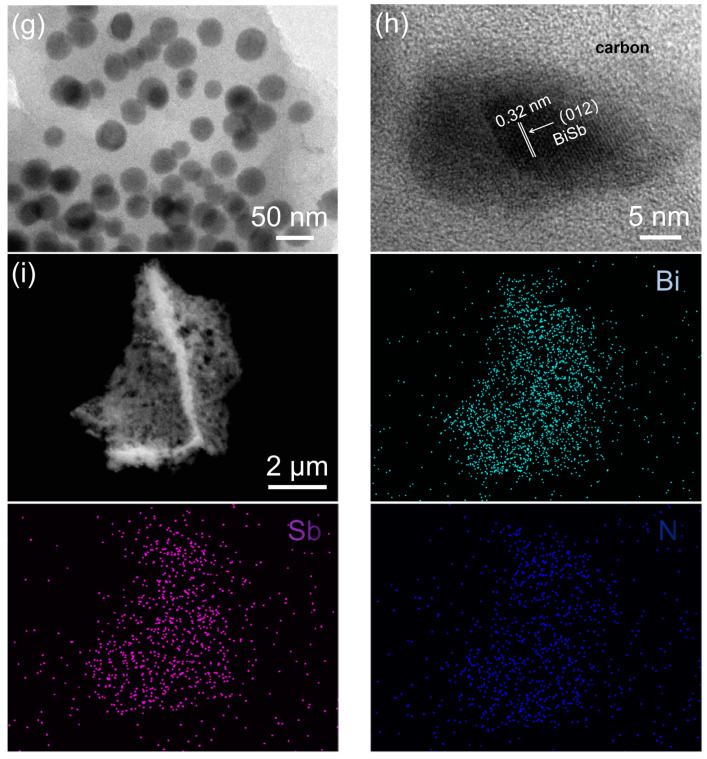
(**a**) Digital photograph and (**b**) SEM image of the PAN-Bi(NO_3_)_3_-SbCl_3_ precursor film; (**c**–**e**) SEM, (**f**,**g**) TME, and (**h**) HRTEM images of BiSb@CSF-0.5; (**i**) elemental mapping images of BiSb@CSF-0.5.

The PAN-Bi(NO_3_)_3_-SbCl_3_ precursor film is depicted in Figure 2a, appearing pure white as a result of the removal of PS. This precursor film possesses high flexibility. Its 3D skeleton structure is revealed in the SEM image (Figure 2b). Upon calcination, the white film is transformed into black film. A low-magnification SEM image (Figure 2c) indicates that BiSb@CSF still maintains the 3D skeleton structure. Most of the macropores are intact. The pore size is 5–20 μm. The formation of macropores is attributed to the large PS colloidal particles. The thickness of the skeleton’s wall ranges from 1 μm to 6 μm. Intriguingly, the skeleton consists of smaller 3D porous skeleton networks, as demonstrated by the SEM image (Figure 2d). This structure is further confirmed by the high-magnification SEM image in Figure 2e as well as the corresponding TEM image in Figure 2f. It can be seen that in the skeleton wall, many carbon fibers are connected together to construct small 3D skeleton networks. The carbon fibers have a diameter of about 100–500 nm. The interwoven carbon fibers create numerous pores. The pore diameter ranges from tens to hundreds of nanometers. These small pores are attributed to small PS colloidal particles. The hierarchical porous skeleton structure can facilitate electrolyte penetration and fast Na^+^ transport. The TEM image (Figure 2g) discloses that the Bi-Sb alloy nanoparticles are evenly embedded inside the carbon fibers. These nanoparticles are approximately spherical. Their diameter ranges from 20 nm to 40 nm. The alloy particles separate from each other. The existence of Bi-Sb alloy is further supported by the HRTEM image. In Figure 2h, a nanoparticle shows a clear lattice fringe with a d-spacing of 0.32 nm, matching the (012) characteristic planes of Bi-Sb alloy. It can be seen that the alloy nanoparticle is tightly surrounded by amorphous carbon. The EDS elemental mappings from SEM (Figure 2i) further confirm the presence and homogenous distribution of Sb, Bi, and N elements within a 3D skeleton fragment. Notably, the signals of Sb and Bi overlap, indicating that the nanoparticles encapsulated in the 3D carbon skeleton exist in the form of Bi-Sb alloy. N is also uniformly doped in the carbon skeleton. EDS patterns and the corresponding element contents of BiSb@CSF-0.5 are shown in Figure S2.

For comparison, Figure S3a shows SEM image of BiSb@DCF. Carbon particles with a size of ~2 μm are assembled in a dense film. There are several very large pores in the film about 10 mm in diameter. This is because the dissolved PS is bound together during the drying process. A high-magnification SEM image (Figure S3b) indicates that Bi-Sb alloy nanoparticles are embedded in the carbon particles. Figure S4a shows a SEM image of BiSb@CCB. Many cubic hollow nanoboxes can be seen. The size of the nanoboxes ranges from 3 μm to 10 μm. The wall thickness of the nanoboxes is 200–500 nm. The high-magnification SEM image (Figure S4b) verifies that BiSb alloy nanoparticles with a size of 70–90 nm are embedded in the wall of the nanobox. To prepare BiSb@CCB, NaCl cubes are used as the template. The surface of the NaCl cubes is coated with a viscous DMF solution dissolved with PAN, Bi(NO_3_)_3_, and SbCl_3_. After drying and calcination, PAN-Bi(NO_3_)_3_-SbCl_3_ is decomposed into a carbon shell embedded with Bi-Sb alloy nanoparticles, and the cubic shape of the NaCl template is well inherited. The NaCl template is then removed with water.

**Figure 3:**
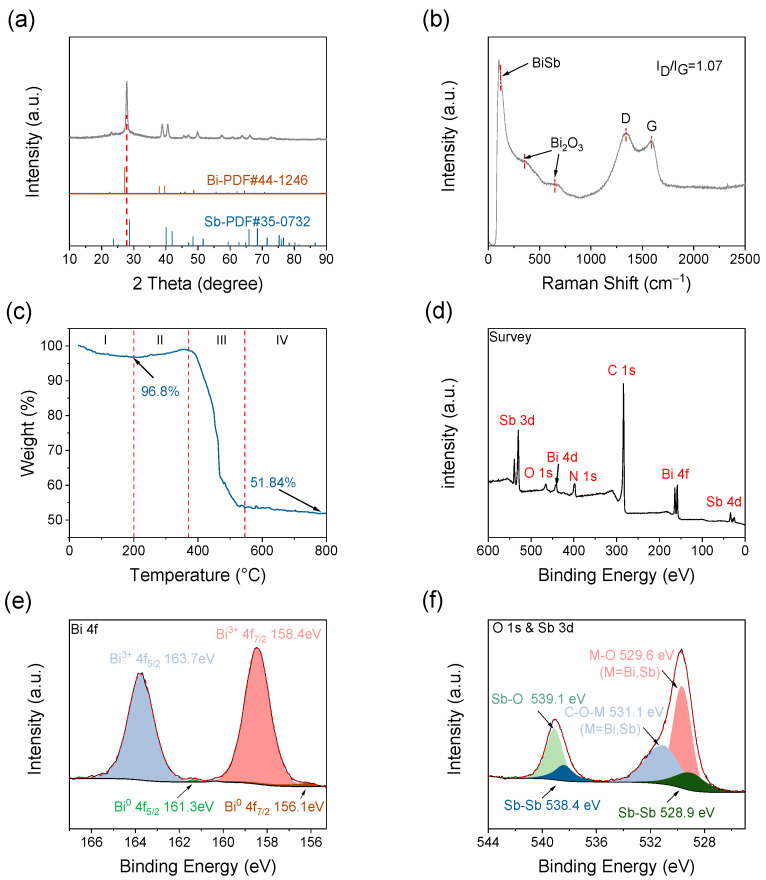

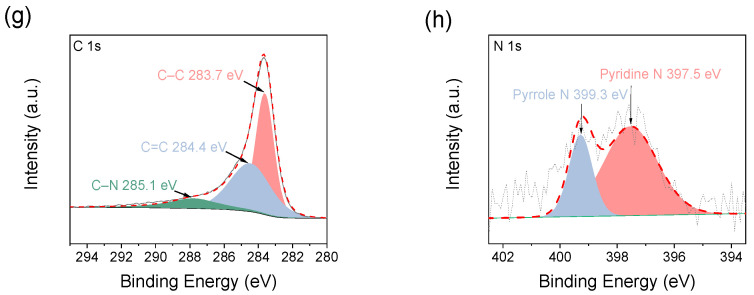
(**a**) XRD pattern of BiSb@CSF-0.5; (**b**) Raman spectrum of BiSb@CSF-0.5; (**c**) TGA curve of BiSb@CSF-0.5 in air; (**d**) XPS survey spectrum of BiSb@CSF-0.5; high-resolution XPS spectra of (**e**) Bi 4f, (**f**) O 1s and Sb 3d, (**g**) C 1s, and (**h**) N 1s.

Figure 3a shows the X-ray diffraction (XRD) pattern of BiSb@CSF-0.5. The diffraction patterns of Bi (JCPDS no. 44-1246) and Sb (JCPDS no. 35-0732) are similar because Bi and Sb have a similar crystal structure (rhombohedral, R-3m) [31,35], but their diffraction angles are different. The XRD pattern of BiSb@CSF is similar to that of Bi and Sb, and there are no other new peaks. However, all the diffraction peaks are located in the middle of the standard diffraction peaks of Bi and Sb. This can be attributed to the contraction of the Bi lattice caused by the replacement of certain Bi atoms by Sb atoms with a smaller radius. The lattice parameters of Bi-Sb alloy are calculated to be a = b = 4.44 Å and c = 11.58 Å, which is similar to the reported value of Bi_1_Sb_1_ alloy [36] based on the refined XRD pattern (Figure S5). The average particle size of Bi-Sb alloy is 34.5 nm, calculated by the Scherrer equation (D = kλ/βcosθ). In the Scherrer equation, D is the average particle size, θ is the diffraction angle, and β is the half-height width of the diffraction peak. λ and k represent the X-ray wavelength and the constant, respectively. The diffraction angle of the strongest peak is 27.8°. The corresponding interplanar spacing, calculated using Bragg’s equation (d = nλ/2sinθ), is determined to be 3.2 Å. This value is consistent with the HRTEM result. The XRD patterns of BiSb@DCF and BiSb@CCB are similar to that of BiSb@CSF, as shown in Figure S6. The Raman spectrum is collected to further characterize the structure of BiSb@CSF. In Figure 3b, the two characteristic peaks at 1348 and 1597 cm^−1^ can be assigned to the D band (sp^3^-C, disordered carbon) and G band (sp^2^-C, graphite carbon), respectively, which indicates that the PAN is decomposed to carbon. The high-intensity ratio of the D and G bands (I_D_/I_G_ = 1.07) suggests that the graphitization of PAN-derived carbon is relatively low. In other words, there are many disordered structures in carbon skeleton matrix. In addition, a shoulder peak appears at ~117 cm^−1^, corresponding to the mixed crystal of Bi-Sb alloy [32]. The other two peaks at 346 and 647 cm^−1^ are derived from Bi_2_O_3_ [32].

The content of Bi-Sb alloy in BiSb@CSF-0.5 is determined by the thermogravimetric analysis (TGA) under flowing air at 10 °C min^−1^ (Figure 3c). A slight weight loss of about 3.20% is observed from room temperature to 200 °C, which is due to the evaporation of absorbed moisture. From 200 °C to 370 °C, the weight increases. This is attributed to the oxidation of Bi-Sb alloy to Bi_2_O_3_ and Sb_2_O_3_. The significant weight loss from 370 °C to 542 °C is caused by the combustion of the carbon skeleton in air. The subsequent weight remains stable. As a result, the content of the Bi-Sb alloy and the carbon skeleton is calculated to be 45.27% and 51.53%, respectively.

X-ray photoelectron spectroscopy (XPS) is used to analyze the element composition and chemical valence of BiSb@CSF. The full survey spectrum (Figure 3d) confirms the presence of Bi, Sb, C, N, and O elements in the BiSb@CSF composite film. Quantitative analysis reveals that the atomic ratio of Bi to Sb is 1:0.90, which is close to the molar ratio (1:1) of the raw material. During the material synthesis process, a small amount of Sb is washed away with water. The high-resolution XPS spectrum of Bi 4f (Figure 3e) can be deconvoluted to four peaks. The two weak peaks centered at 161.3 and 156.1 eV are associated with Bi^0^ 4f_5/2_ and Bi^0^ 4f_7/2_, respectively, representing the Bi-Bi bond. The two strong peaks at 163.7 and 158.4 eV correspond to Bi^3+^ 4f_5/2_ and Bi^3+^ 4f_7/2_, respectively, which are assigned to the Bi-O bond. This indicates that there is a small amount of Bi_2_O_3_ on the surface of Bi-Sb alloy nanoparticles [37]. The high-resolution spectrum of O 1s and Sb 3d (Figure 3f) shows two strong peaks at 539.1 and 529.6 eV, respectively. They are related to Sb-O and M-O (M = Bi, Sb) bonds, respectively. The peak at 531.1 eV corresponds to the C-O-M (M = Bi, Sb) bond, suggesting the formation of oxygen-bridge covalent bonds between the alloy nanoparticle and the carbon skeleton [34]. C-O-M enhances the bonding strength between the Bi-Sb alloy and the carbon skeleton. Furthermore, peaks at 528.9 and 538.4 eV belong to the Sb-Sb bond. The presence of oxides in BiSb@CSF is because the sample is exposed to air and metallic Bi and Sb are oxidized by air. However, the XRD pattern does not show any sign of metal oxides, indicating that the oxides are negligible and only exist on the surface of the Bi-Sb alloy nanoparticles. The C 1s high-resolution spectrum (Figure 3g) is deconvoluted to three peaks at 283.7, 284.4, and 285.1 eV. The three peaks coincide with C-C, C=C, and C-N bonds, respectively [38]. The high-resolution XPS spectrum of N 1s (Figure 3h) can be fitted to two peaks at 399.3 and 397.5 eV, representing pyrrole N and pyridine N, respectively [39]. The atomic percentage of N is calculated to be 7.11%. This implies the formation of N-doped carbon. The doping of N can induce numerous defects and increase the conductivity of the carbon skeleton. Overall, the XPS results demonstrate that Bi-Sb alloy with an atomic ratio close to 1:1 has been synthesized and intimately coupled with a 3D carbon skeleton through strong chemical bonds.

**Figure 4:**
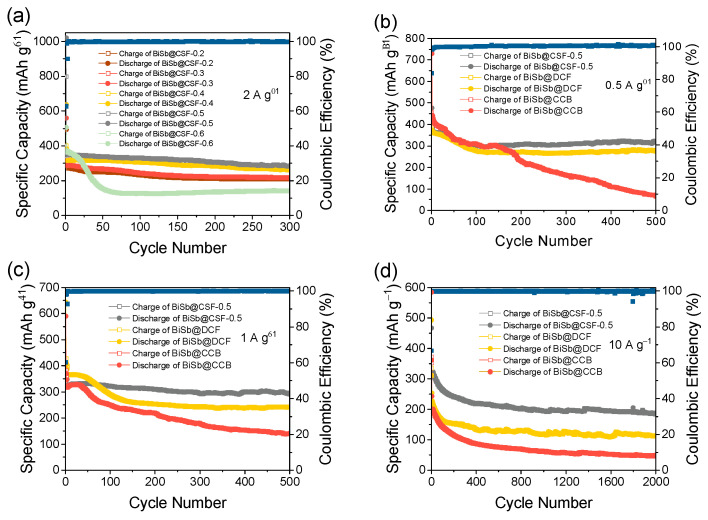

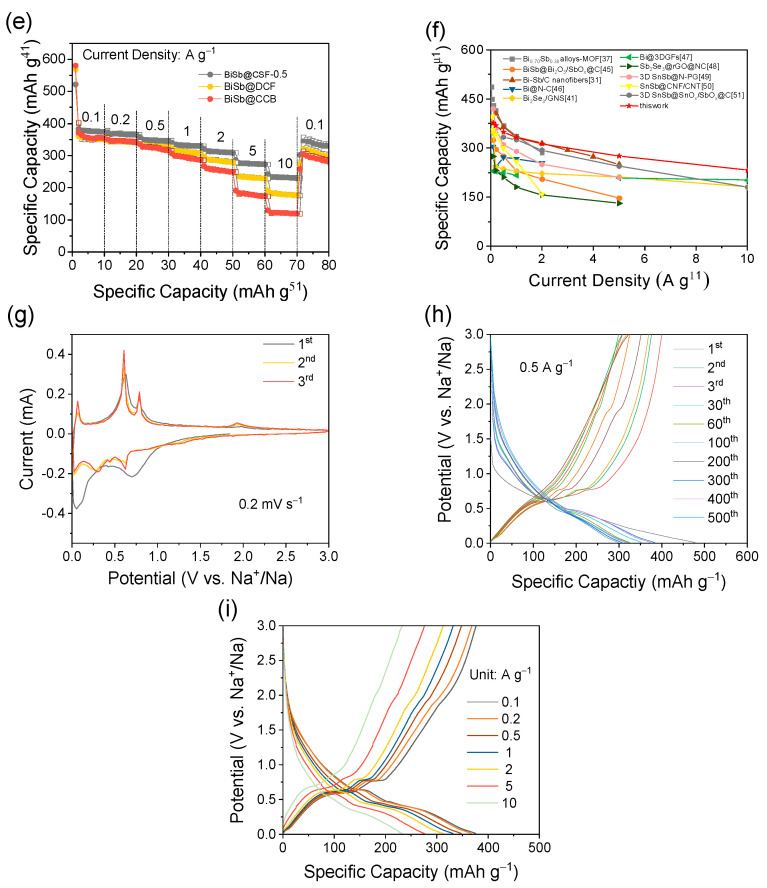
(**a**) Cycling performance of BiSb@CSF with different Bi-Sb alloy contents; comparison of cycling performance of BiSb@CSF-0.5, BiSb@DCF, and BiSb@CCB at current densities of (**b**) 0.5 A g^−1^, (**c**) 1 A g^−1^, and (**d**) 10 A g^−1^; (**e**) rate performance of BiSb@CSF-0.5, BiSb@DCF, and BiSb@CCB from 0.1 to 10 A g^−1^; (**f**) comparison of the rate performance of BiSb@CSF-0.5 with other published metals and alloys for SIB anodes; (**g**) CV curves of BiSb@CSF-0.5 at 0.2 mV s^–1^ in the potential range of 0.01–3.0 V; (**h**) galvanostatic discharge/charge curves of BiSb@CSF-0.5 at 0.5 A g^−1^ in different cycles; (**i**) galvanostatic discharge/charge curves of BiSb@CSF-0.5 at different currents.

Next, the electrochemical properties of BiSb@CSF composites as SIB anodes are systematically investigated with CR2025 half-cells in a potential window of 0.01–3.0 V vs. Na^+^/Na. BiSb@CSF with different Bi-Sb alloy content is first evaluated at 2 A g^−1^ (Figure 4a). When Bi(NO_3_)_3_/SbCl_3_ increases from 0.2/0.2 mmol to 0.3/0/3, 0.4/0/4, and 0.5/0.5 mmol during the material synthesis process, the obtained BiSb@CSF composite exhibits stable and increasing reversible capacity. At the 300th cycle, the discharge capacities reach 210, 218, 262, and 286 mAh g^−1^, respectively. However, when Bi(NO_3_)_3_/SbCl_3_ is further increased to 0.6/0.6 mmol, the discharge capacity of BiSb@CSF rapidly decreases in the first 50 cycles and then stabilizes at ~134 mAh g^−1^. This indicates that the hierarchical porous carbon skeleton film can maintain a robust structure over a relatively wide range of Bi-Sb alloy content. When Bi(NO_3_)_3_/SbCl_3_ is 0.5/0.5 mmol and the corresponding alloy content is 45.27%, the specific capacity and the cycling stability of the composite film are the best. To clarify the structural superiority of BiSb@CSF, BiSb@CSF-0.5, as the representative, is further compared with BiSb@DCF and BiSb@CCB. At 0.5 A g^−1^ (Figure 4b), the discharge capacities of the three composites rapidly decline in the first 100 cycles. The main reason is that the severe volume expansion of Bi-Sb alloy nanoparticles gives rise to their pulverization. In subsequent cycles, BiSb@CSF-0.5 and BiSb@DCF become stable, whereas BiSb@CCB continues declining. At the 500th cycle, the discharge capacities of BiSb@CSF-0.5, BiSb@DCF, and BiSb@CCB are 322, 276, and 68 mAh g^−1^, respectively. At 1 A g^−1^ (Figure 4c), BiSb@CSF-0.5 maintains the most stable and the highest discharge capacity after 50 cycles, whereas BiSb@DCF first decreases and then remains stable. BiSb@CCB keeps declining. At the 500th cycle, BiSb@CSF-0.5, BiSb@DCF, and BiSb@CCB deliver discharge capacities of 292, 243, and 141 mAh g^−1^, respectively. The long-term cycling performance of the three composites is further studied under an ultra-high-current density of 10 A g^−1^. As shown in Figure 4d, BiSb@CSF-0.5, BiSb@DCF, and BiSb@CCB show similar performance trends, but the differences among them become more significant. After 2000 cycles, BiSb@CSF-0.5 provides a discharge capacity of 185 mAh g^−1^ and the capacity decay is only 0.02% per cycle relative to the second discharge. In contrast, the discharge capacities of BiSb@DCF and BiSb@CCB are only 60% and 27% of BiSb@CSF-0.5, respectively. Compared with the conventional cubic carbon nanobox, the carbon film prepared by the spreading method can effectively improve the structural stability and electronic conductivity of Bi-Sb alloy, which is attributed to its unique integrated composite structure. Therefore, BiSb@DCF is superior to BiSb@CCB. Compared with the dense film, the 3D hierarchical porous carbon skeleton structure can enhance electrolyte accessibility, promote Na^+^ transport, increase reaction kinetics, and buffer internal stress. Therefore, BiSb@CSF-0.5 exceeds BiSb@DCF.

The rate capabilities of BiSb@CSF-0.5, BiSb@DCF, and BiSb@CCB are displayed in Figure 4e. The average discharge capacities of BiSb@CSF-0.5 are 391, 367, 349, 333, 313, 277, and 233 mAh g^−1^ at current densities of 0.1, 0.2, 0.5, 1, 2, 5, and 10 A g^−1^, respectively. The high capacity reveals the high electrochemical activity of BiSb@CSF-0.5. It is noteworthy that even if the current is increased to 100-fold, BiSb@CSF-0.5 still maintains 59.6% of its discharge capacity, which demonstrates the excellent rate capability of BiSb@CSF-0.5. When the current is returned to 0.1 A g^−1^, more than 85% reversible capacity (334 mAh g^−1^) can be recovered, disclosing the outstanding structural stability of BiSb@CSF-0.5. Importantly, the reversible capacity of BiSb@CSF-0.5 is much higher than those of BiSb@DCF and BiSb@CCB. The higher the current density, the more significant the superiority of BiSb@CSF-0.5. At 10 A g^−1^, the average capacities of BiSb@DCF and BiSb@CCB are only 78% and 52% of BiSb@CSF-0.5, respectively. This difference is attributed to the fact that the continuous carbon film is more conducive to electron transport. The unique 3D hierarchical porous skeleton structure can further promote electrolyte penetration and Na^+^ transport while effectively buffering the volume change of the Bi-Sb alloy. In other words, BiSb@CSF has higher electronic conductivity and faster reaction kinetics. Moreover, the rate performance of BiSb@CSF-0.5 is better than that of most Bi-Sb composites reported in recent literature, as show in Figure 4f. This confirms outstanding rate performance of BiSb@CSF-0.5.

To figure out the sodiation/desodiation process of BiSb@CSF, the cyclic voltammogram (CV) curves of BiSb@CSF-0.5 are recorded in the initial three cycles at 0.2 mV s^−1^ (Figure 4g). The first cathodic scan shows a broad peak at 0.70 V. This peak disappears in the second and third cycles. This peak is referred to as the formation of solid–electrolyte interphase (SEI) film. The subsequent cathodic peak at 0.05 V is attributed to the sodiation of the carbon skeleton. The first anodic scan shows a weak peak at 0.07 V, which is related to the desodiation of the carbon skeleton. The two strong anodic peaks appear at 0.61 and 0.79 V, corresponding to the two-step dealloying processes (Na_3_BiSb → NaBiSb + 2Na^+^ + 2e^−^, NaBiSb → BiSb + Na^+^ + e^−^, respectively) [37,40]. The two reactions are reversible. The corresponding alloying reactions occur at 0.31 and 0.63 V in the second and third cathodic scans, respectively. Furthermore, a weak anodic peak at ~1.95 V is related to a pseudocapacitive effect, which is associated with ether-based electrolytes [34]. After the first cathodic scan, the almost overlapping CV curves disclose the high stability and reversibility of BiSb@CSF-0.5.

Figure 4h displays the galvanostatic charge/discharge curves of BiSb@CSF-0.5 at 0.5 A g^−1^ in different cycles. The first charge and discharge capacities are 400 and 477 mAh g^−1^. The initial coulombic efficiency (CE) is 83.8%. The capacity loss is attributed to the formation of an SEI layer and the irreversible consumption of Na ions. After the third cycle, the CE is consistently above 98%, demonstrating good reversibility for Na^+^ storage. The charge and discharge curves can maintain similar shapes with the increase in the cycle and basically overlap after the 100th cycle. This demonstrates good cyclic stability. In Figure 4i, the galvanostatic charge/discharge curves of BiSb@CSF-0.5 at different currents are shown. These curves exhibit two distinct discharge plateaus at 0.64 and 0.46 V as well as two obvious charge plateaus at 0.78 and 0.61 V, which correspond to the two-step alloying and dealloying processes, respectively. Even at higher currents of 5 and 10 A g^−1^, the two-plateau feature is still evident, disclosing the excellent rate performance of BiSb@CSF-0.5. In addition, the charge and discharge curves also present an inconspicuous plateau at ~0.06 V, which can be ascribed to the sodiation/desodiation of the carbon skeleton. However, this potential plateau disappears at 5 and 10 A g^−1^, indicating that the Na storage of the carbon skeleton is inhibited. Consequently, at large currents, the Na storage of the composite is mainly contributed to by the Bi-Sb alloy.

**Figure 5:**
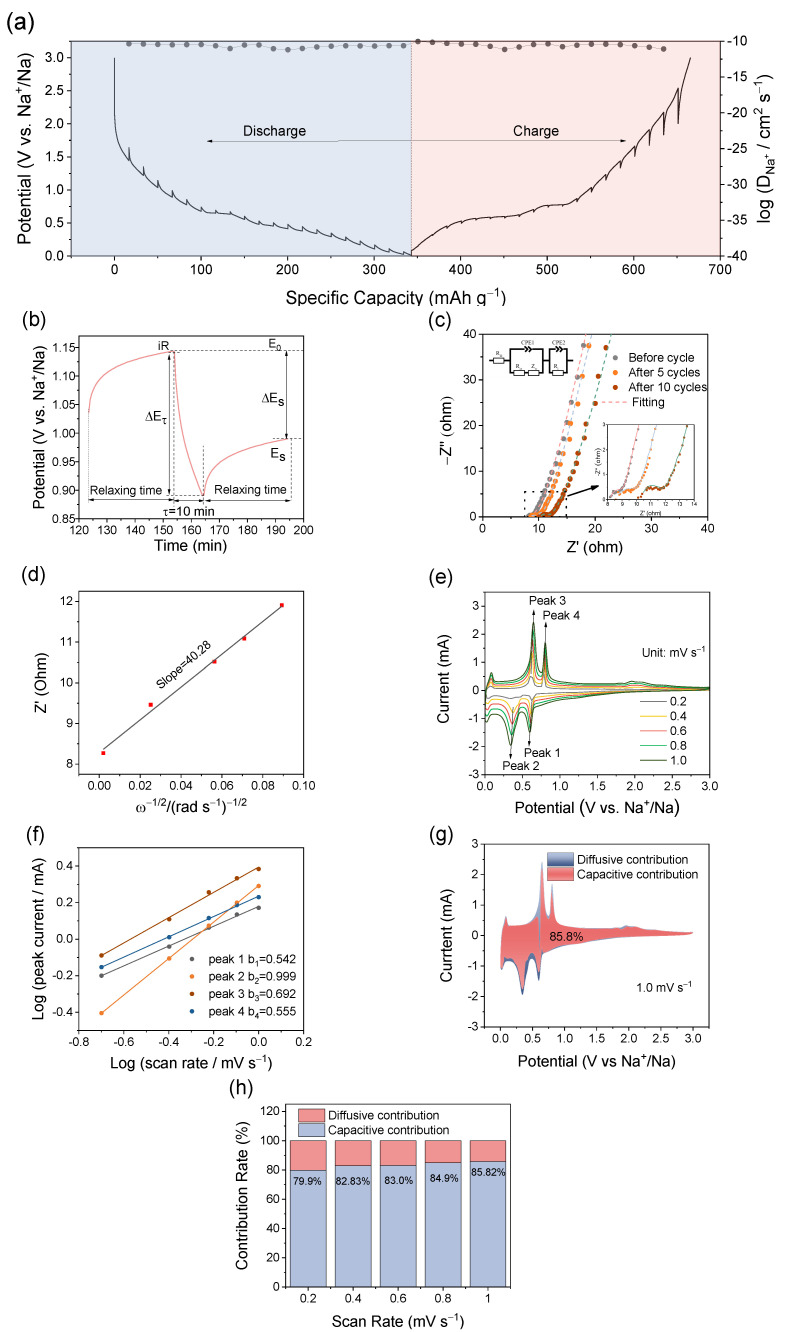
(**a**) GITT curve of BiSb@CSF-0.5 and the Na^+^ diffusion coefficient as a function of the discharging and charging process; (**b**) single GITT titration profile; (**c**) Nyquist plots of BiSb@CSF-0.5 before cycling and at the 5th and 10th cycles; (**d**) relationship between real resistance (Z′) and the angular frequency (ω^−1/2^); (**e**) CV curves of BiSb@CSF-0.5 at scan rates of 0.2, 0.4, 0.6, 0.8, and 1.0 mV s^−1^; (**f**) logarithmic relationship of the peak current and scan rate of CV curves; (**g**) CV curve at 1.0 mV s^−1^ divided into diffusive current and capacitive current; (**h**) normalized capacitive contribution at 0.2, 0.4, 0.6, 0.8, and 1 mV s^−1^.

In order to ascertain the electrochemical mechanism responsible for the superior Na storage performance of BiSb@CSF, systematic kinetic measurements are conducted. The galvanostatic intermittent titration technique (GITT) curves of BiSb@CSF-0.5 are investigated to analyze the diffusion behavior of Na^+^ (Figure 5a). The Na^+^ diffusion coefficient (D) is calculated from the GITT curve based on Fick’s second law (D = 4/πτ × (m_B_V_m_/M_B_S)^2^ × (ΔE_s_/ΔE_τ_)), where τ represents the duration of the current pulse (s) and ΔE_s_ denotes the equilibrium voltage change (V) caused by the current pulse. ΔE_τ_ represents the voltage change (V) during constant current pulse and the iR drop is eliminated. ΔE_s_ and ΔE_τ_ are defined in a single-step GITT curve (Figure 5b). S and M_B_ represent the contact area (cm^2^) of electrolyte–electrode and molar mass (g mol^−1^), respectively. V_m_ and m_B_ correspond to the molar volume (cm^3^ mol^−1^) and the actual mass (g) of the electrode material, respectively. The GITT curve of BiSb@CSF-0.5 exhibits low overpotential, which reflects low polarization and fast kinetics. During the sodiation/discharging and desodiation/charging processes, the Na^+^ diffusion coefficient is relatively stable, ranging from 10^−10^ to 10^−11.2^ cm^2^ s^−1^. The fast Na^+^ diffusion is due to the 3D hierarchical porous skeleton structure and small size of the Bi-Sb alloy nanoparticles. The former facilitates the penetration of the electrolyte, whereas the latter shortens the diffusion distance of Na^+^.

Electrochemical impedance spectroscopy (EIS) is performed on BiSb@CSF-0.5 to reveal its charge transport kinetics (Figure 5c). The Nyquist plots are fitted with a classical equivalent circuit model [7,32,41]. CPE1 and CPE2 represent the constant phase elements. The intersection point of the Nyquist plot on the real axis stands for the internal resistance (R_Ω_). The depressed semicircles in the high- and middle-frequency regions represent solid electrolyte interphase resistance (R_i_) and charge transfer resistance (R_ct_). From the fresh electrode to the 5th and 10th cycles (0.5 A g^−1^), R_Ω_ first stabilizes at 8.2 Ω and then rises to 9.2 Ω. R_ct_ increases from 0.7 Ω to 3.1 Ω and 4.2 Ω. This is attributed to the repeated volume change of Bi-Sb alloy nanoparticles, which loosens the original tight composite structure of BiSb@CSF-0.5. This coincides with the decline in reversible capacity of BiSb@CSF-0.5 at 0.5 A g^−1^ in the first 100 cycles. The oblique line in the low-frequency region indicates the solid-state diffusion of Na^+^ within the electrode material (Warburg impedance, Z_w_). The parallel oblique lines suggest stable Na^+^ diffusion. The diffusion efficiency of Na^+^ can be further calculated on the basis of the Nyquist plot in the low-frequency region. The calculation equations include D = 0.5 (RT/2An^2^F^2^Cσ)^2^ and Z′ = R_s_ + R_ct_ + σω^−1/2^, where T is thermodynamic temperature (298 K). F and R denote Faraday’s constant (96,500 C mol^−1^) and the ideal gas constant (8.314 J K^−1^ mol^−1^), respectively. A denotes the electrode area (cm^2^). n represents the number of transferred electrons. C is the Li^+^ concentration (mol L^−1^). σ is the Warburg coefficient, equal to the slope of the Z′ (the real resistance) vs. ω^−1/2^ (the inverse square root of angular frequency) plot. The fitting result of σ is shown in Figure 5d. The diffusion coefficient of Na^+^ is calculated to be 1.7 × 10^−11^ cm^2^ s^−1^, which is very close to the result of GITT. The fast Na diffusion kinetics contribute to the excellent rate performance of BiSb@CSF-0.5.

CV measurements are performed at different scan rates to shed light on the sodiation/desodiation mechanism of BiSb@CSF-0.5. As can be seen from Figure 5e, the CV curves of BiSb@CSF-0.5 retain similar shapes from 0.2 to 1.0 mV s^−1^. The peak potential shifts slightly, revealing very low polarization. The relationship between the peak current (i_p_) and the scan rate (v) is described as a power–law relationship (i_p_ = av^b^) [42]. a and b are constants. The slope of the linearly fitted log(i_p_)~log(v) plot is considered to be the value of b. The b-value can qualitatively illuminate the charge storage mechanism of the electrode material. The electrochemical reaction is an ideal capacitive process when b approaches 1.0. The ion diffusion process controls the electrochemical reaction when b approaches 0.5. The fitting result of log(i_p_) against log(v) for BiSb@CSF-0.5 is shown in Figure 5f. The four groups of peaks show perfect linear correlations. The b-values are 0.542 (Peak 1), 0.999 (Peak 2), 0.692 (Peak 3), and 0.555 (Peak 4). These b-values indicate that the alloying/dealloying reaction of Na_3_BiSb ↔ NaBiSb is dominated by a strong capacitive process, while the alloying/dealloying reaction of NaBiSb ↔ BiSb is controlled by a weak capacitive process. Furthermore, the capacitive current and diffusive current can be separated from the CV curve based on the following equation (i = k_1_v + k_2_v^1/2^) [18,43]. k_1_v denotes the capacitive current, whereas k_2_v^1/2^ represents the diffusive current. k_1_ and k_2_ are variables related to the potential. The slope and intercept of the linearly fitted i(V)/v^1/2^ vs. v^1/2^ plots correspond to the values of k_1_ and k_2_. The CV curve at 1.0 mV s^−1^ is decomposed into the capacitive capacity and the diffusive capacity, and the capacitive capacity accounts for 85.8% (Figure 5g). For scan rates of 0.2, 0.4, 0.6, and 0.8 mV s^−1^, the capacitive capacities are calculated to be 79.9%, 82.8%, 83.0%, and 84.9%, respectively (Figure 5h). This indicates that the surface capacitance effect controls the charge storage process of BiSb@CSF-0.5, especially at high rates. The capacitive behavior endows BiSb@CSF-0.5 with excellent rate capability.

**Figure 6:**
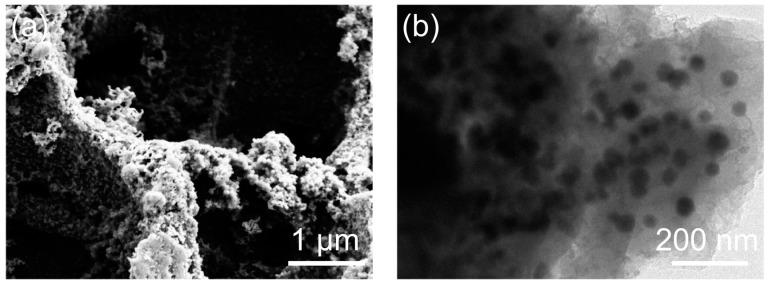
(**a**) SEM and (**b**) TEM images of the cycled BiSb@CSF-0.5 at 1 A g^−1^ after 200 cycles.

BiSb@CSF-0.5 is compared with recently reported Bi-Sb alloy and Bi-based and Sb-based composites, as shown in Table 1. The cycling performance of BiSb@CSF-0.5 is superior to most literature data. Such outstanding performance is closely related to the robust composite structure, which is confirmed by the cycled BiSb@CSF-0.5 electrode. Figure 6a presents an ex situ SEM image of the BiSb@CSF-0.5 electrode at 1 A g^−1^ after 200 cycles. The 3D hierarchical porous skeleton is well preserved and structural collapse does not occur. From the TEM image (Figure 6b), it is evident that the Bi-Sb alloy nanoparticles are still uniformly embedded within the carbon skeleton matrix, certifying that the 3D integrated carbon skeleton enhances the cycling stability of the Bi-Sb alloy nanoparticles.

Based on the aforementioned analysis and discussion, it can be concluded that 3D hierarchical porous carbon skeleton film can significantly improve the electrochemical performance of Bi-Sb alloy for Na storage, and BiSb@CSF-0.5 shows the optimal comprehensive performance. This is mainly attributed to three aspects. (1) The 3D carbon skeleton of BiSb@CSF promotes electron transport, disperses Bi-Sb alloy nanoparticles, prevents the aggregation of alloy nanoparticles, mitigates internal stress, and improves the cycling stability of Bi-Sb alloy by virtue of the robust integrated structure. (2) Hierarchical pores of BiSb@CSF provide a large number of channels for electrolyte infiltration and Na^+^ transport, enlarge the contact area between the electrolyte and the electrode material, and buffer the volume change of the active material during the repeated sodiation/desodiation process. (3) The small Bi-Sb alloy nanoparticles decrease the diffusion distance of Na^+^.

## 3. Experimental

### 3.1. Material Synthesis

Synthesis of BiSb@CSF. A total of 0.35 g PS (M_W_ = 104.14, Aldrich, St. Louis, MI, USA) was dissolved in 5.5 mL N, N-dimethylformamide (DMF). Bi(NO_3_)_3_·5H_2_O and SbCl_3_ were added to the above DMF solution and magnetically stirred for 30 min. A total of 0.25 g PAN (M_W_ = 150,000, Aldrich) was then added. After stirring for 12 h, the white viscous solution was spread on a glass plate with a spreader. Afterwards, water was sprayed onto the precursor solution film to solidify the film. The film was then dried at 100 °C, torn off from the glass plate, and finally calcined at 750 °C for 2 h at a heating rate of 3 °C min^−1^ under an Ar atmosphere. BiSb@CSF was obtained. By changing Bi(NO_3_)_3_·5H_2_O/SbCl_3_ to 0.2/0.2, 0.3/0/3, 0.4/0/4, 0.5/0.5, and 0.6/0.6 mmol, the content of Bi-Sb alloy in BiSb@CSF was adjusted. The corresponding products were denoted as BiSb@CSF-0.2, BiSb@CSF-0.3, BiSb@CSF-0.4, BiSb@CSF-0.5, and BiSb@CSF-0.6, respectively.

Synthesis of BiSb@DCF. The synthesis process was similar to that of BiSb@CSF. The difference was that the precursor solution film was directly dried at 60 °C.

Synthesis of BiSb@CCB. Ten grams NaCl were dissolved in 30 mL deionized water. The pH value of the solution was adjusted to 1.0 with 0.5 M HCl solution. One gram F127 was dissolved in 250 mL ethanol. The NaCl solution was then poured into the F127 solution. The products were separated by vacuum filtration and then calcined at 400 °C for 4 h to obtain NaCl cubes. A total of 0.25 g PAN was dissolved in 5.5 mL DMF. In total, 0.5 mmol Bi(NO_3_)_3_·5H_2_O and 0.5 mmol SbCl_3_ were added into the DMF solution. After stirring for 30 min, 2 g as-prepared NaCl cubes were added and stirred for 12 h. Afterwards, the solution was directly dried overnight at 80 °C. The products were calcinated at 750 °C for 2 h at a heating rate of 3 °C min^−1^ under an Ar atmosphere. The calcined products were repeatedly washed with deionized water to remove the NaCl template.

### 3.2. Material Characterizations

A Rigaku D/max 2500 X-ray diffractometer equipped with Cu Kα radiation (λ = 1.5405 Å) was used to characterize the phase and crystal structure of the as-prepared samples. The Raman spectrum was collected with a Raman spectrometer (Horiba Jobin–Yvon, HR800, LabRam, Villeneuve d’Ascq, France). Thermogravimetric analysis (TGA) was performed with a NETZSCH thermogravimetric analyzer. The elements and their chemical environments were characterized by X-ray photoelectron spectroscopy (XPS, Thermo Scientific K-Alpha spectrometer with Al Kα radiation, Waltham, MA, USA). A scanning electron microscope (SEM, S-4800, Brno-Kohoutovice, Czech Republic) and a transmission electron microscope (TEM, JEM-2100F, Tokyo, Japan) were employed to probe the microstructures and morphologies of the as-prepared materials. An energy dispersive X-ray (EDX, Karlsruhe, Germany) analyzer coupled with SEM was used to analyze the chemical components and elemental mapping of the sample.

### 3.3. Electrochemical Measurements

The electrochemical performance of the sample as a SIB anode was studied on a CR2025 two-electrode coin cell. The coin cells consisted of a counter electrode (fresh Na metal, 99.99 wt%), a separator (glass fiber filter, GF/F, Whatman, Shanghai, China), a working electrode, and an electrolyte (1 M NaPF_6_ dissolved in DEGDME). They were assembled in an argon-filled glovebox. The slurry of the working electrode was prepared by mixing the active material (80%), carbon black (10%), and polyvinylidene fluoride (10%) in 1-methyl-2-pyrrolidone (NMP) and then uniformly spread onto copper foil with a diameter of 12 mm. The electrode was dried in a vacuum oven at 80 °C for 12 h to remove the NMP and water. A BTS-4008 Neware system (Shenzhen, China) was employed to test the rate performance, galvanostatic charge/discharge cycles, and galvanostatic intermittent titration technique (GITT). For GITT, the cell was charged/discharged at 0.1 A g^−1^ for 10 min and then relaxed for 130 min in every pulse. Using the electrochemical workstation (PARSTAT 2273), the cyclic voltammetry (CV) and the electrochemical impedance spectrum (EIS) were performed. EIS was recorded under an AC voltage amplitude of 5 mV. The frequency ranged from 0.01 Hz to 10^5^ Hz.

## 4. Conclusions

In summary, a facile spreading process and soft template strategy are designed to embed Bi-Sb alloy nanoparticles into 3D hierarchical porous carbon skeleton film. The 3D hierarchical porous carbon skeleton film exhibits good stability over a wide range of Bi-Sb alloy content. BiSb@CSF-0.5 achieves optimal reversible capacity and cycling stability. BiSb@CSF-0.5 is also superior to BiSb@DCF and BiSb@CCB. Impressively, even after 2000 cycles at 10 A g^−1^, BiSb@CSF-0.5 still provides a high discharge capacity of 185 mAh g^−1^. The average capacity decay rate is only 0.02%. The exceptional electrochemical performance of BiSb@CSF-0.5 can be attributed to the 3D hierarchical porous carbon skeleton film. Compared with the cube carbon nanobox, the integrated film significantly improves the structure robustness and electronic conductivity of Bi-Sb alloy. In comparison with dense carbon film, 3D hierarchical porous carbon skeleton further enhances electrolyte accessibility, promotes the contact between electrolyte and electrode material, improves reaction kinetics, and boosts the structure stability. As a result, BiSb@CSF-0.5 is superior to BiSb@DCF, and BiSb@DCF is better than BiSb@CCB. This work cleverly combines Bi-Sb alloy with 3D porous carbon films, offering a novel and promising anode material for advanced SIBs.

## Figures and Tables

**Table 1:** Comparison of cycling performance of BiSb@CSF-0.5 and the reported Bi-Sb and Bi-based and Sb-based composites for SIBs.

Composites	Current Density (mA·g^−1^)	Capacity (mA·g^−1^)	Cycling Number	Ref.
G-Bi_0.33_Sb_0.67_ np-Bi_2_Sb_6_	125	328	1000	[44]
	1000	150	10,000	[40]
Bi_0.70_Sb_0.30_alloys-MOF	200	259.8	500	[36]
	1000	180.9	1000	
BiSb@Bi_2_O_3_/SbO_x_	100	293	100	[45]
	1000	248.4	500	
Bi-Sb/C nanofibers	2000	233.2	2000	[31]
Bi@N-C	1000	307	400	[46]
Bi_2_Se_3_/GNS	2000	222	1000	[41]
	10,000	183	1000	
Bi@3DGFs	100	208	95	[47]
Sb@P-N/C	500	295.6	400	[7]
Sb_2_Se_3_@rGO@NC	200	180	1000	[48]
3D SnSb@N–PG	100	400	100	[49]
	10,000	190	4000	
SnSb@CNF/CNT	500	210	700	[50]
	1000	161	1000	
SnSb@SnO_x_/SbO_x_@C	100	360	200	[51]
	2000	195	500	
BiSb@CSF-0.5	500	323	500	This work
	1000	291	500	
	10,000	186.5	2000	

## Data Availability

The data presented in this study are available on request from the corresponding authors.

## Supplementary Materials

The following supporting information can be downloaded at: https://www.mdpi.com/article/10.3390/molecules28186464/s1, Figure S1: Optical micrographs of (a) PAN-PS-Bi(NO_3_)_3_-SbCl_3_ solution coated on a glass plate, (b) PAN-Bi(NO_3_)_3_-SbCl_3_ film, (c) dried PAN-Bi(NO_3_)_3_-SbCl_3_ film, (d) directly dried PAN-PS-Bi(NO_3_)_3_-SbCl_3_ film; Figure S2: EDS pattern of BiSb@CSF-0.5; Figure S3: SEM images of BiSb@DCF; Figure S4: SEM image of BiSb@CCB; Figure S5: Refined XRD pattern of BiSb@CSF-0.5; Figure S6: XRD patterns of (a) BiSb@DCF and (b) BiSb@CCB.

## Sample Availability

Samples of the compounds are available from the authors.
