# Supplementary material for: Bismuth-Antimony Alloy Nanoparticles Embedded in 3D Hierarchical Porous Carbon Skeleton Film for Superior Sodium Storage
Source: Molecules. 2023 Sep 6;28(18):6464. doi: 10.3390/molecules28186464 (PMC10534634; doi:10.3390/molecules28186464)
Supplement: Supplementary file 1 [file molecules-28-06464-s001.zip › molecules-2578028-supplementary.pdf]

Supplementary Materials for
Bismuth-antimony alloy nanoparticles embedded in 3D hierarchical porous carbon skeleton film for superior sodium storage

Jiafan Wang[1], Yonghui Lin[2], Wei Lv[2], Yongfeng Yuan[1,3*], Shaoyi Guo[1,3*], Weiwei Yan[4]

[1]*College of Machinery Engineering, Zhejiang Sci-Tech University, Hangzhou 310018, China*

[2]*Zhejiang Ecowell Energy Management Technology Co., LTD. Hangzhou, Zhejiang, 310012, China*

[3]*Changshan Research Institute, Zhejiang Sci-Tech University, Changshan 324299, China*

[4]*College of Metrology and Measurement Engineering, China Jiliang University, Hangzhou, 310018, China*

[*] Corresponding author. *E*-mail address: yuanyf@zstu.edu.cn (Y.F. Yuan), syiguo@zstu.edu.cn (S.Y. Guo)

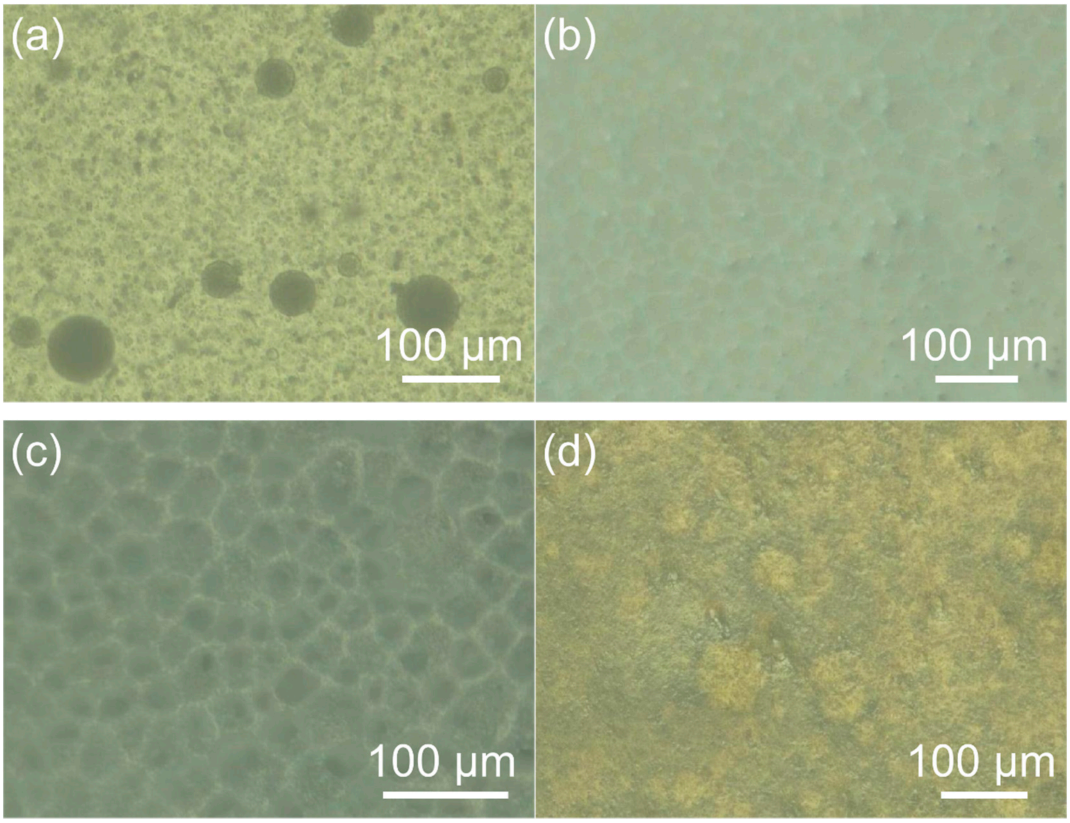

Fig. S1 Optical micrographs of (a) PAN-PS-$Bi(NO_3)_3$-$SbCl_3$ solution coated on a glass plate, (b) PAN-$Bi(NO_3)_3$-$SbCl_3$ film, (c) Dried PAN-$Bi(NO_3)_3$-$SbCl_3$ film, (d) Directly dried PAN-PS-$Bi(NO_3)_3$-$SbCl_3$ film.

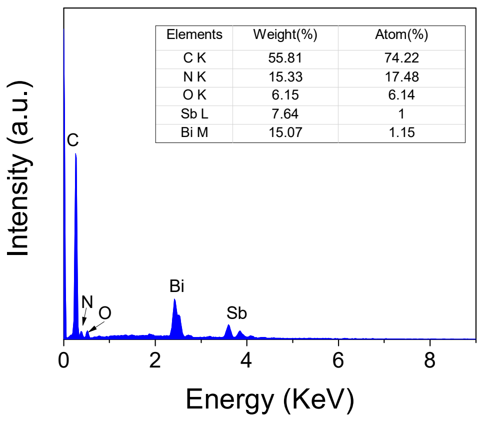

Fig. S2 EDS pattern of BiSb@CSF-0.5.

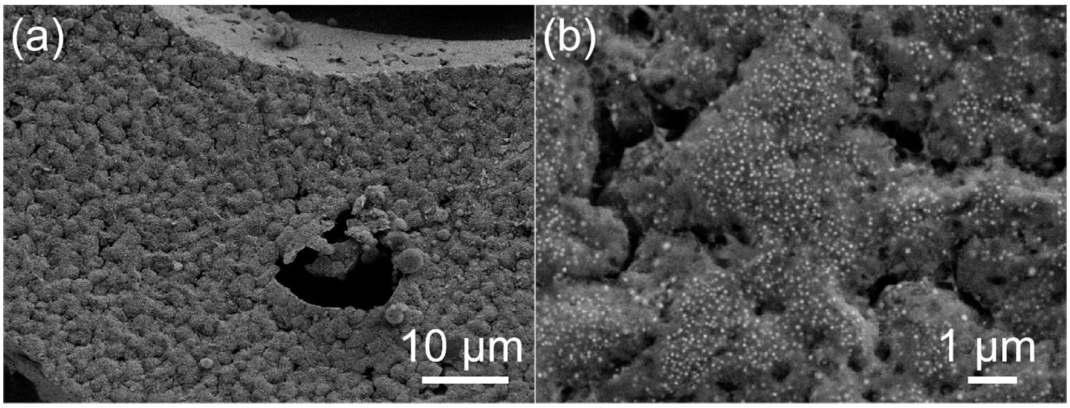

Fig. S3 SEM images of BiSb@DCF.

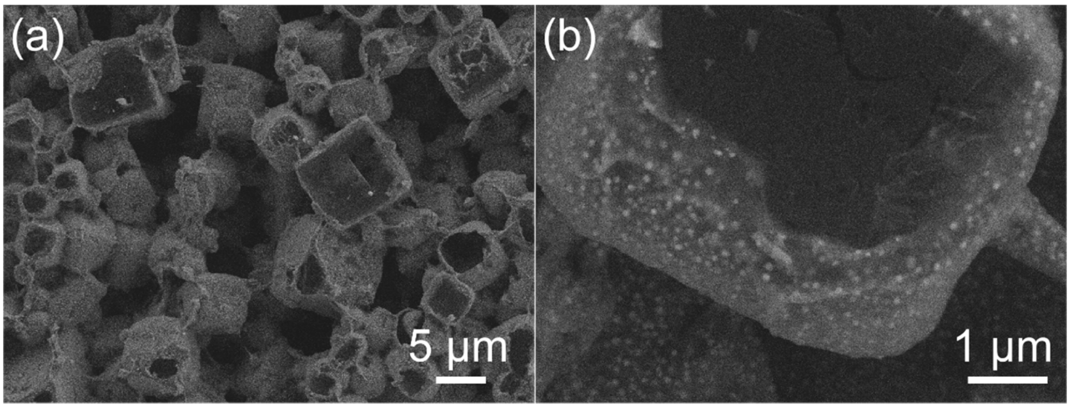

Fig. S4 SEM image of BiSb@CCB.

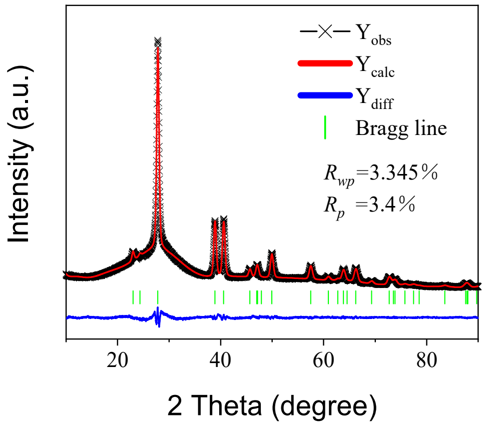

Fig. S5 Refined XRD pattern of BiSb@CSF-0.5.

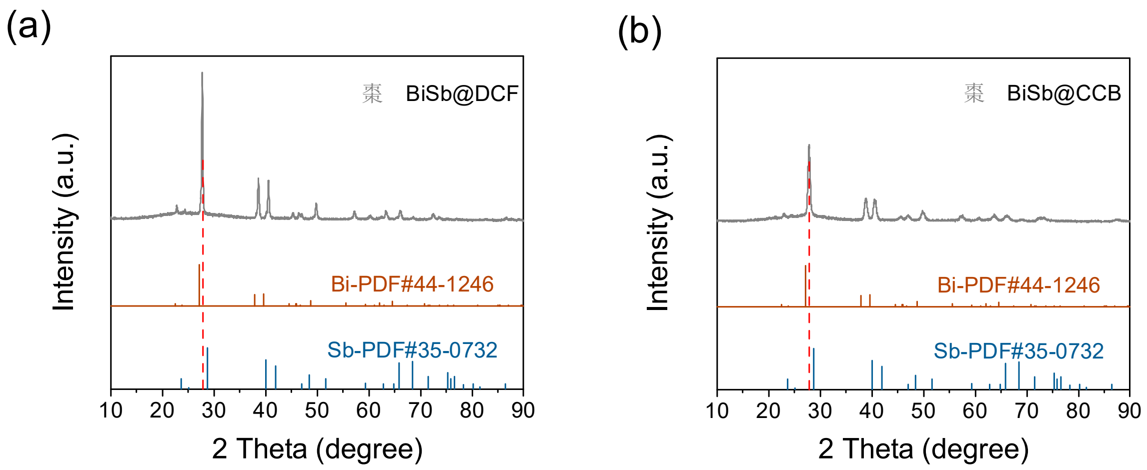

Fig. S6 XRD patterns of (a) BiSb@DCF and (b) BiSb@CCB.
